# State of the art of lower limb prosthesis simulators: A literature review

**DOI:** 10.1017/wtc.2026.10038

**Published:** 2026-03-03

**Authors:** Imke Neelen, Bob van der Windt, Matthew Justin Major, Gerwin Smit

**Affiliations:** 1 Biomechanical Engineering, Delft University of Technology, Netherlands; 2 Institute of automatic control, RWTH Aachen University, Germany; 3 Physical Medicine and Rehabilitation, Northwestern University, USA

**Keywords:** prosthetics, biomechanics, design

## Abstract

Individuals with limb loss present significant challenges to testing and evaluating prosthetic devices, such as medical approval processes and participant availability. Prosthesis simulators, designed for mimicking prosthesis use with able-bodied individuals, offer an alternative to conducting controlled experiments and enhancing the development of prosthetic technologies. This review examines the design features, applications, and limitations of lower limb prosthesis simulators. A literature search identified 73 studies that have used lower limb prosthesis simulators. Most studies have focused on transfemoral prosthesis simulators (TFsims) and testing prosthetic designs and control mechanisms. The most frequently assessed movement was walking, while other movements, were explored only sporadically. The findings reveal significant variability in simulator configurations, training protocols, and the range of movements assessed. Additionally, a notable research gap exists in evaluations of the effect of transtibial prosthesis simulators (TTsims) and hip disarticulation prosthesis simulators (HDsims) on gait. Despite these challenges, prosthesis simulators offer promising potential for accelerating and improving prosthesis development while putting less stress on the relatively small target group of individuals with limb loss. Further research is needed to standardize methodologies and better understand the effects of simulator design and training on gait performance to facilitate advancements in prosthetic research.

## 1. Introduction

### 1.1. Motivation

The population of individuals with limb loss in the United States is expected to increase from 1.6 million in 2005 to 3.6 million in 2050 (Ziegler-Graham et al., 2008). In the Netherlands, approximately 3,300 lower limb amputations are performed every year (Spoorendonk et al., 2020), ranging from hip disarticulations to partial foot amputations. Losing a leg considerably impacts daily life. Prosthetic devices can help restore mobility (Kobayashi et al., 2024). However, user satisfaction remains a challenge, with studies reporting that 40% to 57% of lower limb prosthesis users are dissatisfied with their prosthesis (Dillingham et al., 2001; Berke et al., 2010). One of the primary reasons for the abandonment of prosthetic devices is interface comfort, but another important factor is their functionality. Specifically, 43% of people with a hip disarticulation prosthesis stop using their prosthesis within 20 months of fitting (Ueyama et al., 2020). Difficulty in ambulation is one of the main reasons for abandoning the hip disarticulation prosthesis (Ueyama et al., 2020). In knee, ankle, and foot prostheses, the same motivations for abandonment are observed.

Conventional leg prostheses cannot fully replicate biological function, which leads to inefficient walking and compensatory movements. Leg prosthesis users often walk with slower speeds, shorter stride lengths, and decreased cadence due to the lack of push-off in their prosthetic feet (Schmid-Zalaudek et al., 2022). Prosthesis users have lower ground reaction force (GRF) magnitudes and reduced push-off on the prosthetic side, with the intact side absorbing increased loads during weight-bearing phases. Walking with a prosthesis incurs a higher metabolic cost, particularly in transfemoral prosthesis users, due to mechanical inefficiency in their knee and ankle prostheses and compensatory strategies (Schmid-Zalaudek et al., 2022). Increased joint moments and forces in the intact limb increase the risk of osteoarthritis and overuse injuries (Nolan and Lees, 2000; Gailey et al., 2008).

### 1.2. Challenges in prosthesis design

To enhance prosthesis design and user satisfaction, iterative development and testing are essential. However, conducting research with prosthesis users to evaluate the effects of prosthesis design on user outcomes poses significant challenges. Ethical and medical approval processes, designed to protect individuals with medical conditions such as limb loss, often require extensive documentation and time and, though important, can delay the development cycle. Approval is required for each iteration of a prototype, which limits the speed and flexibility of human testing. Participant recruitment presents an additional challenge. The population of prosthesis users is relatively small, of which only a subset is often available and willing to participate in research. Repeatedly involving the same individuals in multiple rounds of testing places a considerable burden on this group. Furthermore, heterogeneity within this cohort, such as differences in amputation level, cause of limb loss, age, mobility level, and comorbidities, further reduces the number of suitable participants for evaluating a specific type of prosthesis (Smith, 2006).

### 1.3. Prosthesis simulators as a solution

Prosthesis simulators, also called able-bodied adapters, bypass adapters, or bypass orthoses, are designed to mimic prosthesis use with anatomically intact individuals and offer a promising alternative for research, allowing for controlled experimentation, improved understanding of prosthetic gait, and even clinical training (Kobayashi et al., 2024). These simulators enable able-bodied individuals to serve as their own control subjects (Schlafly and Reed, 2021) by fitting a prosthesis to an intact limb, thereby simulating novice prosthesis use. With the use of a prosthesis simulator, shortcomings in the functioning of the novice prosthesis can be detected earlier, leading to improvements in prosthesis design and thereby improving the user satisfaction. Prosthesis simulators are widely employed in research and development. They can mimic the three main levels of lower extremity amputation: an amputation below the knee, a transtibial amputation (Dictionary – ISPO, 2025); an amputation above the knee, a transfemoral amputation (Dictionary – ISPO, 2025); and an amputation through the hip joint, a hip disarticulation (Dictionary – ISPO, 2025). Lower limb prosthesis simulators have been used to evaluate prosthesis design parameters, including volitional control (Zhang et al., 2014), knee flexion strategies (Keeken et al., 2012a), and ankle stiffness (Louessard et al., 2022). While lower limb prosthesis simulators may be a valuable tool in prosthetic research, they may not fully replicate prosthetic gait (Kobayashi et al., 2024). Studies indicate that able-bodied individuals walking with lower limb prosthesis simulators approximate prosthetic gait in several aspects but may differ in walking speed (Lemaire et al., 2000) and vertical GRFs (Kobayashi et al., 2024).

### 1.4. Objectives

Various lower limb prosthesis simulators have been developed for research, each tailored to specific study objectives and amputation levels. Given the diversity of study parameters, researchers have designed custom simulators to meet their needs. The aims of this literature review were to summarize the range of lower limb prosthesis simulator designs implemented in research, describe their specific applications and limitations, and identify unintended effects on gait parameters imposed by use of a simulator that may not reflect prosthetic gait. Findings from this review will provide the prosthetics research community with an overview of the status of prosthesis simulators and their utility for gait studies on prosthetic components distal to the socket and reveal potential design improvements to enhance their ability to replicate prosthetic gait.

## 2. Methods

### 2.1. Search query

From December 1, 2024, till December 31, 2024, a literature search of the databases Scopus, PubMed, and Web of Science was conducted. In accordance with the study aim, we searched for articles that described a lower limb prosthesis simulator without constraints on the type of study or outcomes. Therefore, the following Boolean combination of keywords was used: ((“prosthesis simulator”) OR (“prosthetic simulator”) OR (“dummy prosthesis”) OR (“prosthetic knee simulator”) OR (“training prosthesis”) OR (“bypass prosthesis”) OR (“bypass socket”) OR (“bypass orthosis”)) AND ((lower AND limb) OR (lower AND extremity) OR (leg) OR (knee) OR (ankle) OR (hip) OR (transfemoral) OR (transtibial)). This general search string was adapted for each database. A limiter of the English language was applied to the search, with no restrictions on publication date.

### 2.2. Selection criteria

This review included articles mentioning the implementation of a lower limb prosthesis simulator for any amputation level (partial foot, transtibial, knee disarticulation, transfemoral amputation, hip disarticulation) to test a lower limb prosthesis with an able-bodied individual. Prosthesis simulators for both unilateral and bilateral amputations were included. Articles using virtual reality or augmented reality instead of mechanical simulators were excluded, as were articles using computer model simulations. Articles were also excluded if evaluation with the prosthesis simulator was not included (i.e., when only the design/type of a simulator was described and evaluated in a bench test). First, all the titles and abstracts were scanned for relevance, and then, the full text of relevant articles was evaluated against the selection criteria. For those articles that were ultimately included, the reference list was reviewed according to the same process.

### 2.3. Data extraction

**Figure 1.**
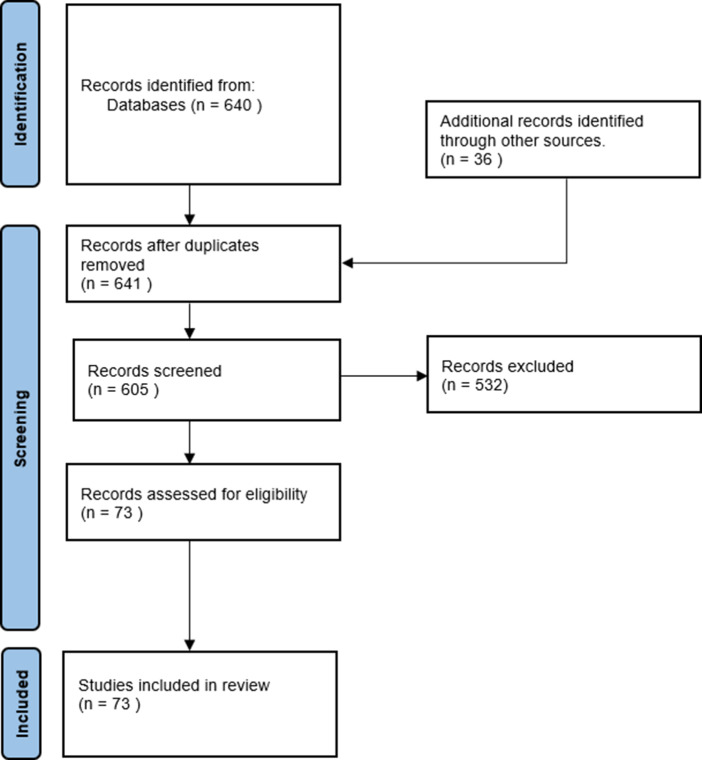
PRISMA flowchart of the literature search process (Page et al., 2021).

The following information was retrieved from each article: authors, year of publication, simulated amputation location, tested prosthetic joint, the aim of testing, anatomical location of simulator (e.g., distal or lateral), additions made to the non-affected leg (e.g., height-adjusted shoe), type of movements tested (e.g., gait, stair walking, etc.), measurements done, attachment type (e.g., how is the amputation simulated), amount and type of training, term used for the prosthesis simulator, mass, material, and whether the tested prosthesis is an active, semi-active, or passive prosthesis. See Figure 1.

## 3. Results

### 3.1. State of the art

A total of 73 articles were reviewed in this study, with publication years between 1973 and 2025. Some articles were published online ahead of print, and thus, the publication year was 2025 despite the review being conducted in 2024.

#### 3.1.1. Overview of prosthesis simulators

**Figure 2.**
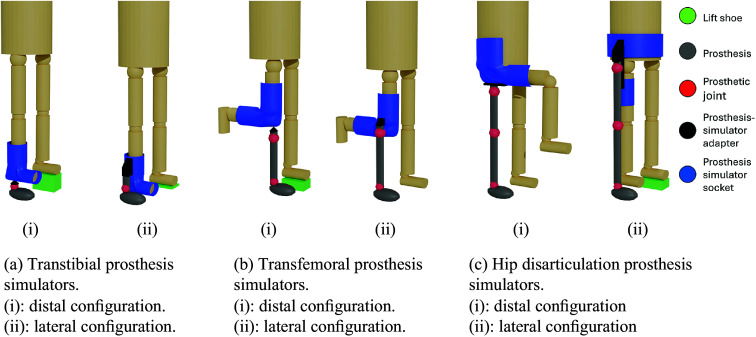
Configuration of the prosthesis simulators. Blue: prosthesis simulator socket; red: prosthetic joints; green: lift shoe; gray: prosthesis; black: adapter. Left side of each subfigure shows the distal configuration, and right side shows the lateral configuration prosthesis.

Three types of lower limb prosthesis simulators were identified: transfemoral prosthesis simulators (TFsims) (Dictionary – ISPO, 2025), transtibial prosthesis simulators (TTsims) (Dictionary – ISPO, 2025), and hip disarticulation prosthesis simulators (HDsims) (Dictionary – ISPO, 2025). No prosthesis simulators were found for partial foot amputations. The studies included in this review configured the prosthesis either distally or laterally relative to the original joint. A distally mounted prosthesis aligns the prosthetic joint with the original joint in the sagittal plane, maintaining the longitudinal alignment of the limb. This distal configuration of a prosthesis simulator is shown in Figure 2a(i), b(i), and c(i). In contrast, a laterally mounted prosthesis aligns with the anatomical joint in the frontal plane, maintaining medial-lateral alignment, as shown in Figure 2a(ii), b(ii), and c(ii). This arrangement represents a lateral configuration of the prosthesis simulator. No commercially available prosthesis simulators were reported in the literature, but commercial devices intended for other purposes (e.g., crutch assistance) were modified to function as (part of) a prosthesis simulator.

#### 3.1.2. Terms

**Figure 3.**
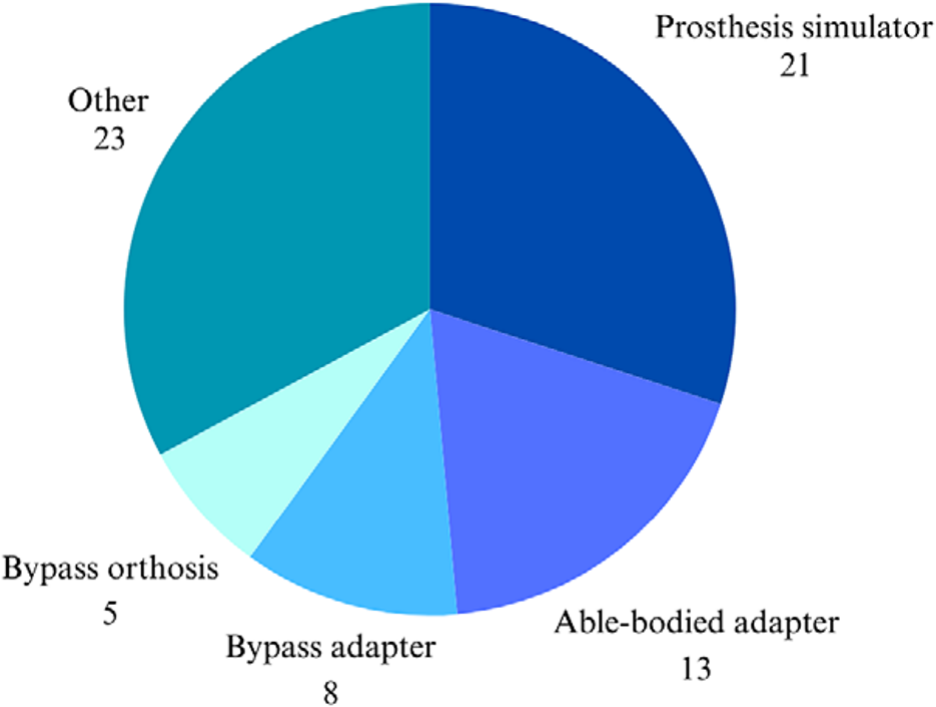
Terms used in the literature to describe prosthesis simulators. “Other” includes terms used less than three times, including “fake socket,” “dummy socket,” and “pseudo-prosthesis”.

Only 37 of 73 included articles were retrieved through the initial search query (the remainder were retrieved from the secondary reference list search), primarily due to the variety of terms used to describe prosthesis simulators. Across the 73 articles, “prosthesis simulator” and variations on that, such as “prosthetic simulator,” were the most frequently used terms in the reviewed literature. See Figure 3 for terms used in the reviewed literature.

#### 3.1.3. Research focus

Thirty-five articles used a prosthesis simulator to test a new control function for a lower limb prosthesis, in some cases in combination with a novel design of a prosthesis. A wide variety of control functions were tested, such as stance stability control, impedance control, myoelectric control, variable stiffness, and neuromuscular impedance control (Flowers, 1973; Fite et al., 2007; Sup et al., 2007, 2008; Varol et al., 2008; Collins and Kuo, 2010; Hoover and Fite, 2010; Waycaster et al., 2010; Hoover and Fite, 2011; Wu et al., 2011; Lawson et al., 2012; Gregg et al., 2013; Liu et al., 2013; Shultz et al., 2013; Lenzi et al., 2014a, b; Horn, 2015; Kim and Collins, 2015; LaPrè and Sup, 2015; Lenzi et al., 2015; Thatte and Geyer, 2015; Zhao et al., 2015; Grimmer et al., 2016; LaPrè et al., 2016; Quintero et al., 2016; Lenzi et al., 2017a, b; Pagel et al., 2017; Andrysek et al., 2019; Thatte et al., 2019; Elery et al., 2020; Ueyama et al., 2020; Best et al., 2021; Zhu et al., 2022; Pranata et al., 2023).

Eleven studies focused on evaluating new prosthesis designs without modifying control functions (Ramakrishnan et al., 2015; Cempini et al., 2017; Schlafly et al., 2017; During et al., 2018; Murabayashi and Inoue, 2020; Schlafly and Reed, 2020, 2021; Murabayashi et al., 2022; Murabayashi and Inoue, 2022; Bader et al., 2023; Mroz et al., 2024).

In addition to the test of new prostheses, 18 experimental studies focused on various topics (Hansen et al., 2000; Adamczyk et al., 2006; Vanicek et al., 2007; Zelik et al., 2011; Keeken et al., 2012a, b; Caputo and Collins, 2014; Zhang et al., 2014; Wentink et al., 2015; Jin et al., 2016; Ramakrishnan et al., 2017; Groothuis and Houdijk, 2019; Kooiman et al., 2020; Embry and Gregg, 2021; Krausz and Hargrove, 2021; Louessard et al., 2022; Pillet et al., 2022; Bonnet-Lebrun et al., 2024), such as kinematic adaptations to novel ambulatory conditions (Vanicek et al., 2007; Kooiman et al., 2020), the effect of knee height on gait asymmetry (Ramakrishnan et al., 2017), and the influence of the replacement of prosthetic foot energy return on metabolic cost (Bonnet-Lebrun et al., 2024).

Several studies uniquely explored alternative applications of prosthesis simulators outside of testing devices or their acute effects on gait quality. A study developed a temporary training prosthesis that was modified for able-bodied subjects for testing (Gholizadeh et al., 2024). Another study focused on designing a TFsim (Oberg et al., 2021), while one assessed a new prosthetic pump for vacuum-assisted suspension (Major et al., 2015), and two investigated algorithms for initial prosthesis fitting and tuning of a prosthesis (Aghasadeghi et al., 2013; Senatore et al., 2023). Only four studies directly examined prosthesis simulators (Lemaire et al., 2000; Champagne, 2017; Fanous et al., 2023; Kobayashi et al., 2024).

### 3.2. Transtibial prosthesis simulators

#### 3.2.1. Type

**Figure 4.**
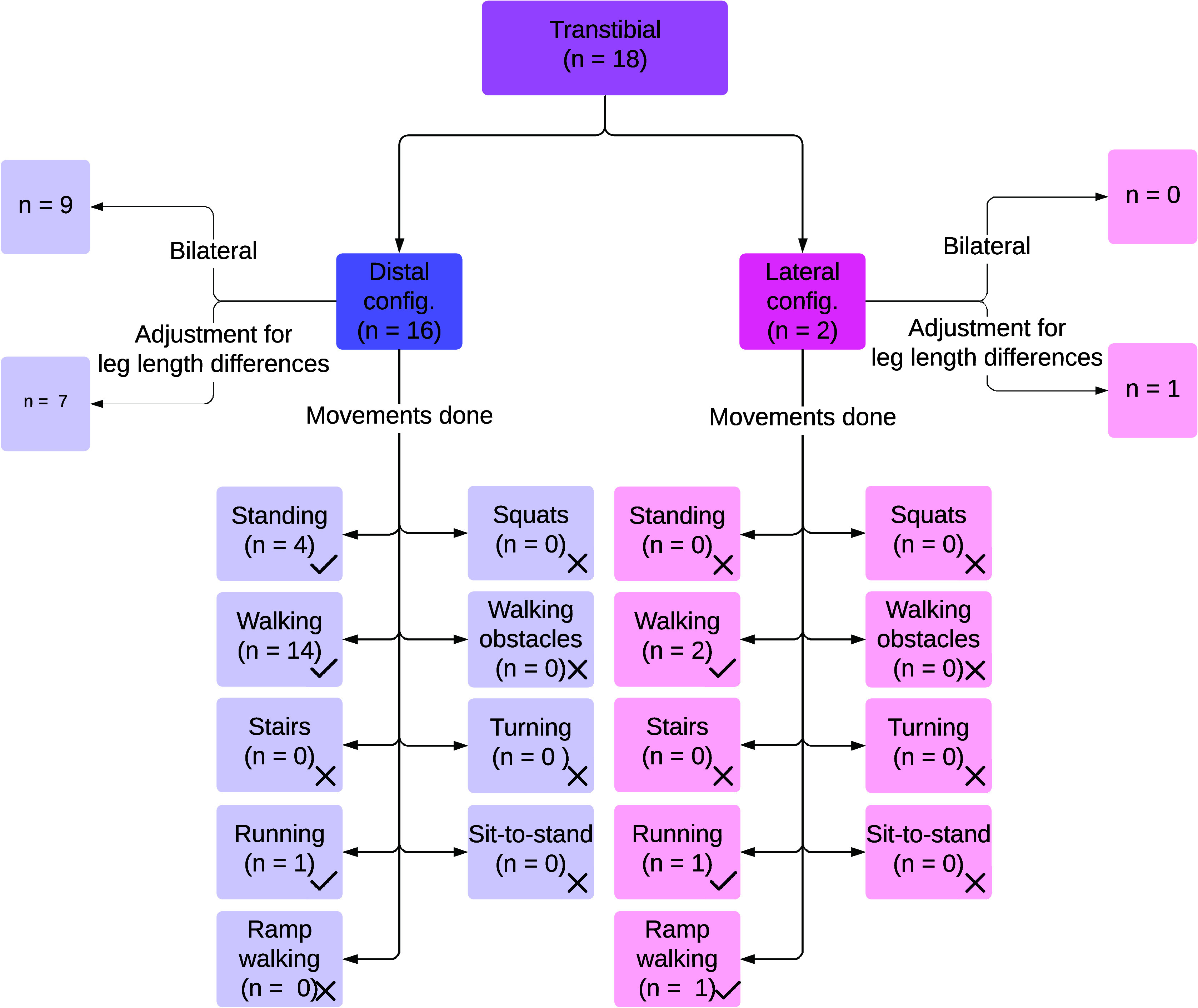
Results on transtibial prosthesis simulators, including configurations of the prosthesis simulators, adjustments on the unaffected leg to compensate for leg length differences caused by prosthesis simulators, and movements done with these prosthesis simulators.

In TTsims, a transtibial amputation (i.e., below the knee amputation) is simulated by immobilizing the ankle joint, typically at approximately 90 degrees, using a boot or an ankle-foot orthosis. Eighteen studies used TTsims (Hansen et al., 2000; Adamczyk et al., 2006; Collins and Kuo, 2010; Zelik et al., 2011; Shultz et al., 2013; Caputo and Collins, 2014; Kim and Collins, 2015; LaPrè and Sup, 2015; Major et al., 2015; Grimmer et al., 2016; Jin et al., 2016; LaPrè et al., 2016; Cempini et al., 2017; Lenzi et al., 2017b; During et al., 2018; Groothuis and Houdijk, 2019; Louessard et al., 2022; Gholizadeh et al., 2024). Figure 4 shows an overview of the reported TTsims and their performance activities studied.

#### 3.2.2. Configuration

Sixteen studies used TTsims with a distal configuration (Figure 2a[i]) (Hansen et al., 2000; Adamczyk et al., 2006; Collins and Kuo, 2010; Zelik et al., 2011; Shultz et al., 2013; Caputo and Collins, 2014; Kim and Collins, 2015; LaPrè and Sup, 2015; Major et al., 2015; Jin et al., 2016; LaPrè et al., 2016; Cempini et al., 2017; During et al., 2018; Groothuis and Houdijk, 2019; Louessard et al., 2022; Gholizadeh et al., 2024), of which nine applied the TTsims bilaterally (Adamczyk et al., 2006; Shultz et al., 2013; LaPrè and Sup, 2015; Major et al., 2015; LaPrè et al., 2016; Cempini et al., 2017; Groothuis and Houdijk, 2019; Hansen et al., 2000; Louessard et al., 2022). The remaining studies adjusted for leg length differences using a lift shoe on the unaffected leg (Collins and Kuo, 2010; Zelik et al., 2011; Caputo and Collins, 2014; Kim and Collins, 2015; Jin et al., 2016; Gholizadeh et al., 2024) or by having the unaffected leg walk over a raised platform (During et al., 2018).

Two studies used TTsims with a lateral configuration (Figure 2a[ii]) (Grimmer et al., 2016; Lenzi et al., 2017b), one of which adjusted for leg length differences using a 1.5 cm insole for the unaffected leg despite a 5 cm leg length increase of the affected leg (Grimmer et al., 2016), while the other did not mention adjustments (Lenzi et al., 2017b).

#### 3.2.3. Performance activities

Walking was tested in 16 studies using TTsims. Both studies with a lateral configuration assessed walking (Grimmer et al., 2016; Lenzi et al., 2017b), with speeds reaching 1.6 m/s (Grimmer et al., 2016). Among studies with a distal configuration, 14 out of 16 studies tested walking (Hansen et al., 2000; Adamczyk et al., 2006; Collins and Kuo, 2010; Zelik et al., 2011; Shultz et al., 2013; Caputo and Collins, 2014; Kim and Collins, 2015; LaPrè and Sup, 2015; Major et al., 2015; Jin et al., 2016; LaPrè et al., 2016; Cempini et al., 2017; During et al., 2018; Gholizadeh et al., 2024), with a maximum speed of 1.5 m/s (Jin et al., 2016). One study using a lateral configuration examined ramp ascent (Lenzi et al., 2017b). Four studies focused on standing tasks using a TTsim with a distal configuration (Hansen et al., 2000; Cempini et al., 2017; Louessard et al., 2022; Gholizadeh et al., 2024). Running was tested in two studies: one with a distal configuration (Groothuis and Houdijk, 2019) and another with a lateral configuration, reaching speeds of 4.0 m/s (Grimmer et al., 2016).

#### 3.2.4. Commercial availability

Three out of the 18 studies used modified commercial products as TTsims. AirCast pneumatic boots (Adamczyk et al., 2006; Collins and Kuo, 2010) and Roller Derby Proline 900 inline skates (Shultz et al., 2013) were used to immobilize the ankle. Both boots were modified with a pyramidal adapter to attach a prosthesis underneath them.

#### 3.2.5. Material and mass

Materials were described in only two articles: plastic (Gholizadeh et al., 2024) and aluminum (Groothuis and Houdijk, 2019; Gholizadeh et al., 2024). Foam padding was used to enhance the comfort of the interface (Gholizadeh et al., 2024).

The mass of TTsims was reported to be up to 1.9 kg (Caputo and Collins, 2014). The AirCast pneumatic boots are reported to be 0.85 kg for the medium-sized and 1.05 kg for the large-sized boot (Adamczyk et al., 2006).

### 3.3. Transfemoral prosthesis simulators

#### 3.3.1. Type

In TFsims, a transfemoral (above-knee) amputation or knee disarticulation is simulated by rendering the knee joint and all distal structures nonfunctional. A total of 51 studies used TFsims. The sources that used TFsims are the following:

(Flowers, 1973; Lemaire et al., 2000; Fite et al., 2007; Vanicek et al., 2007; Sup et al., 2007, 2008; Varol et al., 2008; Hoover and Fite, 2010, 2011; Waycaster et al., 2010; Wu et al., 2011; Keeken et al., 2012a, b; Lawson et al., 2012; Aghasadeghi et al., 2013; Gregg et al., 2013; Liu et al., 2013; Zhang et al., 2014; Lenzi et al., 2014a, b, 2015, 2017a; Horn, 2015; Ramakrishnan et al., 2015; Thatte and Geyer, 2015; Wentink et al., 2015; Zhao et al., 2015; Quintero et al., 2016; 2017; Champagne, 2017; Pagel et al., 2017; Schlafly et al., 2017; Andrysek et al., 2019; Thatte et al., 2019; Elery et al., 2020; Kooiman et al., 2020; Schlafly and Reed, 2020, 2021; Murabayashi and Inoue, 2020, 2022; Best et al., 2021; Embry and Gregg, 2021; Krausz and Hargrove, 2021; Oberg et al., 2021; Zhu et al., 2022; Bonnet-Lebrun et al., 2024; Kobayashi et al., 2024; Murabayashi et al., 2022; Pillet et al., 2022; Pranata et al., 2023; Senatore et al., 2023).

**Figure 5.**
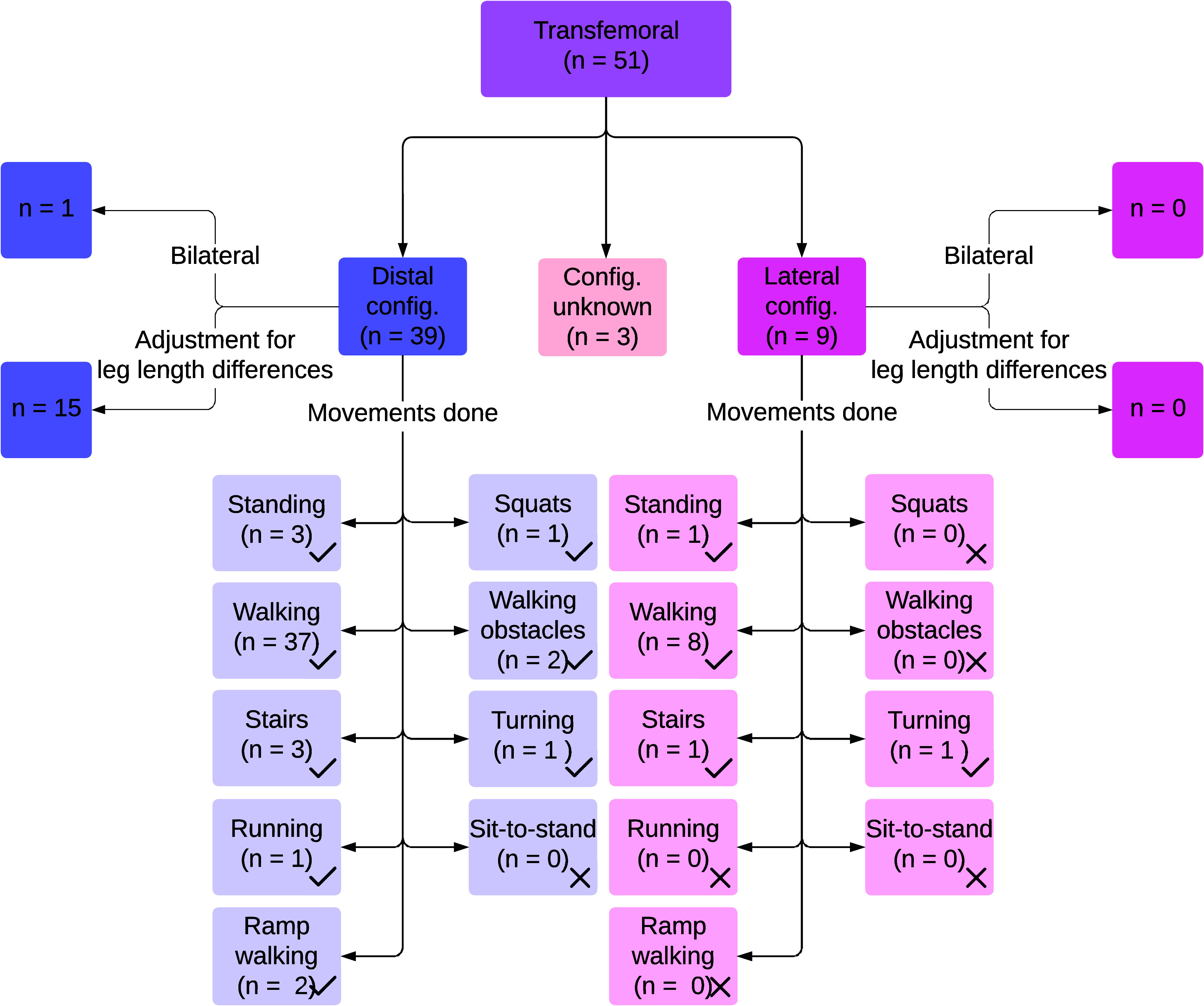
Results on transfemoral prosthesis simulators, including configurations of the prosthesis simulators, adjustments on the unaffected leg to compensate for leg length differences caused by prosthesis simulators, and activities performed with these prosthesis simulators.

Four studies used these simulators to test new ankle-foot or foot components (Schlafly et al., 2017; Schlafly and Reed, 2020, 2021; Pillet et al., 2022). Figure 5 shows an overview of the reported TFsims and their performance activities studied.

Most (48 of 51) TFsims employed an L-shaped design, in which the healthy lower limb is folded backward at approximately 90 degrees. Three studies used a V-shaped design, in which the knee is flexed as much as possible, keeping the lower leg closer to the body (Flowers, 1973; Lemaire et al., 2000; Kobayashi et al., 2024). The inertial differences between a L-shaped and V-shaped socket for TFsims are discussed in Section 3.6.

#### 3.3.2. Configuration

Thirty-nine studies using TFsims employed a distal configuration (Figure 2b[i]) (Lemaire et al., 2000; Vanicek et al., 2007; Keeken et al., 2012a, b; Lawson et al., 2012; Aghasadeghi et al., 2013; Gregg et al., 2013; Liu et al., 2013; Lenzi et al., 2014a, 2015, 2017a; Horn, 2015; Ramakrishnan et al., 2015, 2017; Thatte and Geyer, 2015; Wentink et al., 2015; Zhao et al., 2015; Quintero et al., 2016; Champagne 2017; Pagel et al., 2017; Schlafly et al., 2017; Andrysek et al., 2019; Thatte et al., 2019; Elery et al., 2020; Kooiman et al., 2020; Murabayashi and Inoue, 2020, 2022; Schlafly and Reed, 2020, 2021; Best et al., 2021; Krausz and Hargrove, 2021; Oberg et al., 2021; Murabayashi et al., 2022; Pillet et al., 2022; Zhu et al., 2022; Pranata et al., 2023; Senatore et al., 2023; Bonnet-Lebrun et al., 2024; Kobayashi et al., 2024). Figure 2b[i] presents an example of this distal configuration. Fifteen of these studies using a distal configuration adjusted for leg length differences using a higher sole, often called a lift shoe, on the unaffected leg (Aghasadeghi et al., 2013; Gregg et al., 2013; Liu et al., 2013; Lenzi et al., 2014a; Horn, 2015; Lenzi et al., 2015; Thatte and Geyer, 2015; Zhao et al., 2015; Pagel et al., 2017; Elery et al., 2020; Best et al., 2021; Pranata et al., 2023; Senatore et al., 2023), by modifying prosthesis length (Thatte et al., 2019), or by flexing the unaffected leg in response to a shorter affected limb (Keeken et al., 2012a). One study used bilateral TFsims with a distal configuration (Lawson et al., 2012). The remaining 23 studies did not mention leg length adjustments.

Nine studies used a TFsim in a lateral configuration (Figure 2b[ii]) (Flowers, 1973; Fite et al., 2007; Sup et al., 2007, 2008; Varol et al., 2008; Hoover and Fite, 2010, 2011; Waycaster et al., 2010; Wu et al., 2011), none of which reported leg length adjustments.

Three studies did not mention or show pictures of the location of the simulator, adjustments on the unaffected leg, or about the shape of socket used (Lenzi et al., 2014b; Zhang et al., 2014; Embry and Gregg, 2021).

#### 3.3.3. Performance activities

Walking was the most common (48 of 51) activity study with TFsims. Eight out of nine studies that used a lateral configuration tested walking (Flowers, 1973; Fite et al., 2007; Sup et al., 2007, 2008; Varol et al., 2008; Waycaster et al., 2010; Hoover and Fite, 2011; Wu et al., 2011), with speeds reaching up to 1.2 m/s (Hoover and Fite, 2011).

Walking was assessed in 37 of 39 studies with distal configurations (Lemaire et al., 2000; Vanicek et al., 2007; Keeken et al., 2012b; Lawson et al., 2012; Aghasadeghi et al., 2013; Gregg et al., 2013; Liu et al., 2013; Lenzi et al., 2014a, 2017a; Horn, 2015; Ramakrishnan et al., 2015, 2017; Thatte and Geyer, 2015; Wentink et al., 2015; Zhao et al., 2015; Quintero et al., 2016; Champagne 2017; Pagel et al., 2017; Schlafly et al., 2017; Andrysek et al., 2019; Thatte et al., 2019; Elery et al., 2020; Kooiman et al., 2020; Murabayashi and Inoue, 2020; Schlafly and Reed, 2020, 2021; Best et al., 2021; Krausz and Hargrove, 2021; Oberg et al., 2021; Murabayashi and Inoue, 2022; Murabayashi et al., 2022; Pillet et al., 2022; Zhu et al., 2022; Pranata et al., 2023; Senatore et al., 2023; Bonnet-Lebrun et al., 2024; Kobayashi et al., 2024), with maximum speeds of 1.6 m/s (Elery et al., 2020).

Three studies reported walking, but did not specify the mounting location of the prosthesis (Lenzi et al., 2014b; Zhang et al., 2014; Embry and Gregg, 2021).

Several studies included additional movement conditions. Two studies, both using a distal configuration, added obstacles during walking: one simulated tripping by applying extra torque to the knee prosthesis (Thatte and Geyer, 2015), while another study required subjects to step on 3 cm-high blocks on a treadmill (Thatte et al., 2019). One study investigated running at 2 m/s and squatting at a frequency of 0.25 Hz (15 squats per minute) with a distal configuration, in addition to walking (Zhu et al., 2022). Four articles using a TFsim tested ramp walking, both ascending and descending, with maximum slopes of 8 degrees (Zhang et al., 2014; Best et al., 2021; Embry and Gregg, 2021; Krausz and Hargrove, 2021). Two of the studies used a distal configuration (Best et al., 2021; Krausz and Hargrove, 2021), while the mounting location was unspecified in the others (Zhang et al., 2014; Embry and Gregg, 2021). Two studies examined turning in place, one with a distal configuration (Krausz and Hargrove, 2021) and one with a lateral configuration (Varol et al., 2008). Both of these also evaluated standing still on the prosthesis, as did two additional studies with a distal configuration (Keeken et al., 2012a, b).

Stair climbing with a TFsim was examined in four studies: three tested stair ascent (Hoover and Fite, 2010; Lenzi et al., 2015, 2017a), while one assessed both ascent and descent (Krausz and Hargrove, 2021). In one of these articles, a lateral configuration was used and the height of the stair steps was reported as 18 cm (Hoover and Fite, 2010). The other three studies used a distal configuration and did not report the height of the stairs.

#### 3.3.4. Commercial availability

The iWalk crutch (iWALKFree, 2025) was used as a TFsim in five articles (Thatte and Geyer, 2015; Champagne, 2017; Schlafly et al., 2017; Schlafly and Reed, 2021; Senatore et al., 2023). Three of these studies modified the crutch to attach a prosthesis (Thatte and Geyer, 2015; Schlafly et al., 2017; Schlafly and Reed, 2021). One study assessed kinematics during walking with the iWalk crutch itself without a distal prosthetic component (Champagne, 2017) and one used the iWalk for an experimental protocol (Senatore et al., 2023).

#### 3.3.5. Material and mass

The materials described were fiberglass (Hoover and Fite, 2010, Hoover and Fite, 2011, plastic (Vanicek et al., 2007; Lawson et al., 2012), and aluminum (Vanicek et al., 2007; Hoover and Fite, 2011; Lawson et al., 2012). Foam paddings are used for interface comfort (Hoover and Fite, 2011) and Velcro straps to mount the simulator to the participant anatomy (Hoover and Fite, 2010). The TFsim from the oldest article was made from a leather harness and a molded socket, but the molding material was not described (Flowers, 1973). None of the articles using a TFsim described the mass of the simulator.

### 3.4. Hip disarticulation prosthesis simulators

#### 3.4.1. Type

**Figure 6.**
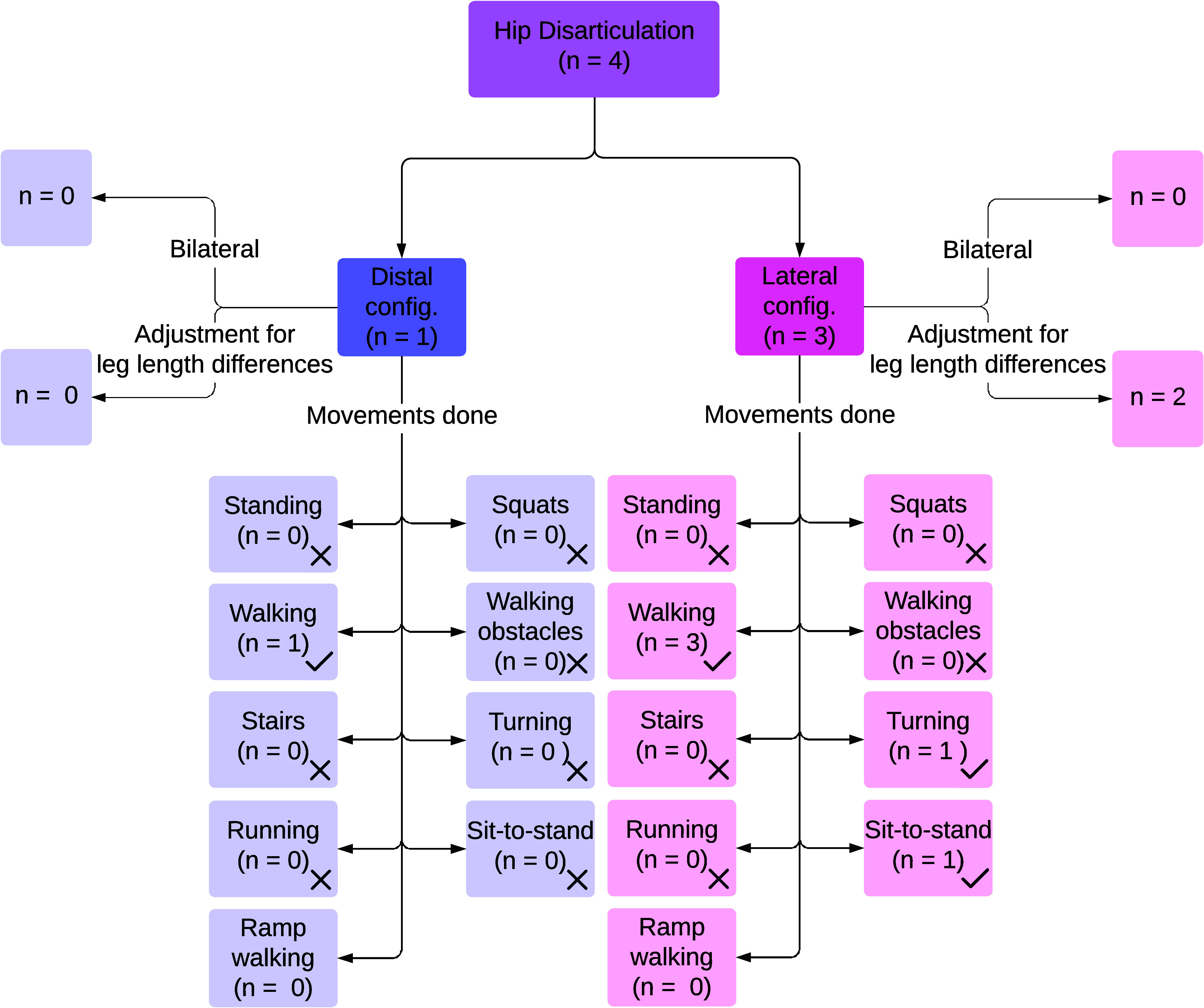
Results on hip disarticulation prosthesis simulators, including mounting positions of prostheses, adjustments on the unaffected leg to compensate for leg length differences caused by prosthesis simulators, and movements done with these prosthesis simulators.

In HDsims, a full removal of the leg at the level of the hip joint is simulated by disabling the function of the hip joint and all distal structures. Four studies implemented HDsims (Ueyama et al., 2020; Bader et al., 2023; Fanous et al., 2023; Mroz et al., 2024). Figure 6 shows an overview of the reported HDsims and their performance activities studied.

#### 3.4.2. Configuration

Of the four studies using HDsims, one used a distal configuration (Figure 2c[i]) (Ueyama et al., 2020) without mentioning leg length adjustments. The remaining three used a lateral configuration (Figure 2c[ii]) (Bader et al., 2023; Fanous et al., 2023; Mroz et al., 2024), two of which employed a lift shoe on the unaffected leg (Fanous et al., 2023; Mroz et al., 2024), while one configuration flexed the affected leg to prevent toe drag (Bader et al., 2023) while the prosthesis pointed outwards laterally.

#### 3.4.3. Performance activities

All four studies using HDsims assessed walking (Ueyama et al., 2020; Bader et al., 2023; Fanous et al., 2023; Mroz et al., 2024), with reported speeds ranging from 0.33 m/s (Fanous et al., 2023) to 0.69 m/s (Mroz et al., 2024). Two articles both tested with three participants, walking with either one or two canes (Bader et al., 2023; Mroz et al., 2024). In one study, the participant walked without an assistive device (Ueyama et al., 2020), and another study did not specify (Fanous et al., 2023) whether an assistive device was used.

Only one study evaluated movements beyond steady level walking. In addition to a 2-minute walk test, participants completed the L-test, which involves standing up from a chair, walking, turning, and sitting down again (Fanous et al., 2023).

#### 3.4.4. Commercial availability

For the hip prosthesis simulator, a hip abduction orthosis from Orthomerica (Orthomerica Products, Inc., 2025) was modified to attach the hip prosthesis (Fanous et al., 2023).

#### 3.4.5. Material and mass

The prosthesis simulators for hip disarticulations were made from a plaster cast (Ueyama et al., 2020) or a commercially available hip abduction orthosis (Fanous et al., 2023) reinforced with carbon fiber (Ueyama et al., 2020; Fanous et al., 2023) and acrylic resin (Fanous et al., 2023).

The HDsim with a distal configuration had a socket made from plaster cast with a mass of 1.5 kg (Ueyama et al., 2020). For a HDsim with a lateral configuration, the hip prosthesis was connected to the orthosis by an adapter. The first reported version had a weight of 2.7 kg, which was reduced to 1.2 kg using a lighter material following participant feedback (Fanous et al., 2023).

### 3.5. Training and accommodation

In several studies, subjects were allowed to accommodate to the prosthesis simulator and associated prosthesis conditions prior to experiments. The extent of accommodation training varies widely across the reviewed articles and therefore categorized according to the amount of training as reported from least to most.

#### 3.5.1. No training

Four articles (Vanicek et al., 2007; Major et al., 2015; Kooiman et al., 2020; Oberg et al., 2021) provided no training, in which the authors clearly stated that the subjects received no accommodation time. Two studies tested adaptations to novel situations, where a lack of prior simulator experience was necessary (Vanicek et al., 2007; Kooiman et al., 2020). One study designed a TFsim for which participants provided feedback (Oberg et al., 2021), and one study used the simulator to assess a prosthesis under simulated operational loading and therefore no participant training was necessary (Major et al., 2015).

#### 3.5.2. Minimal training

Studies that were classified as providing minimal training either tuned and fitted the prosthesis while using the simulator prior to experiments or provided an acclimation period, without any explicit goals for this training. Twelve articles were included in this category (Fite et al., 2007; Sup et al., 2007, 2008; Varol et al., 2008; Wu et al., 2011; Zelik et al., 2011; Keeken et al., 2012b; Gregg et al., 2013; Lenzi et al., 2015; Jin et al., 2016; Quintero et al., 2016; Andrysek et al., 2019) .

#### 3.5.3. Well trained

Studies classified as including “well trained” participants delivered accommodation until a certain goal was achieved, such as a satisfactory speed, ambulation without assistance, or until comfortable. Ten articles were included in this category (Adamczyk et al., 2006; Hoover and Fite, 2010; LaPrè and Sup, 2015; Wentink et al., 2015; Champagne 2017; Pagel et al., 2017; Ramakrishnan et al., 2017; Embry and Gregg, 2021; Krausz and Hargrove, 2021; Fanous et al., 2023; Bonnet-Lebrun et al., 2024; Mroz et al., 2024).

#### 3.5.4. Experienced

Five articles included participants who were experienced users of prosthesis simulators, albeit without description of that level of experience (Lenzi et al., 2014b; Kim and Collins, 2015; Ramakrishnan et al., 2015; Best et al., 2021; Bader et al., 2023).

#### 3.5.5. Thoroughly trained

The category with the most training is “thoroughly trained,” in which the subjects had specific training sessions prior to the experiment, often on multiple days, sometimes guided by a physical therapist. Seventeen articles assessed participants who were thoroughly trained prior to data testing (Lemaire et al., 2000; Lawson et al., 2012; Liu et al., 2013; Shultz et al., 2013; Caputo and Collins, 2014; Zhang et al., 2014; Groothuis and Houdijk, 2019; Thatte et al., 2019; Elery et al., 2020; Murabayashi and Inoue, 2020; Schlafly and Reed, 2020; Ueyama et al., 2020; Schlafly and Reed, 2021; Murabayashi et al., 2022; Pillet et al., 2022; Senatore et al., 2023; Kobayashi et al., 2024). Training ranged from 1 hour (Schlafly and Reed, 2020, 2021) in a single session, to 10 hours, separated over multiple days (Ueyama et al., 2020), up to experience with the TFsim over 6 months (Kobayashi et al., 2024).

#### 3.5.6. Unknown

For 23 articles (Flowers, 1973; Hansen et al., 2000; Collins and Kuo, 2010; Waycaster et al., 2010; Keeken et al., 2012a; Aghasadeghi et al., 2013; Lenzi et al., 2014a, 2017a, b; Horn, 2015; Thatte and Geyer, 2015; Zhao et al., 2015; Grimmer et al., 2016; LaPrè et al., 2016; Cempini et al., 2017; During et al., 2018; Louessard et al., 2022; Murabayashi and Inoue, 2022; Zhu et al., 2022; Pranata et al., 2023; Gholizadeh et al., 2024), no accommodation or training was described, but also it was not explicitly stated that no training was provided.

### 3.6. Effects of transfemoral prosthesis simulators

Of the 73 articles included in this literature review, 51 used TFsims; therefore, this section focuses on the effects of TFsims in gait. While most articles used a prosthesis simulator in accordance with the level of amputation the prosthesis was designed for, four articles chose to test an ankle-foot prosthesis with a TFsim, implementing a prosthetic knee joint as well (Schlafly et al., 2017; Schlafly and Reed, 2020, 2021; Pillet et al., 2022).

Two studies that evaluated gait in subjects using TFsims by comparing kinematic, kinetic, and spatiotemporal parameters to those of amputees walking with a prosthesis (Lemaire et al., 2000; Kobayashi et al., 2024) reported that able-bodied subjects using a TFsim walked significantly slower than prosthesis users, as reported in the literature. However, kinetic and kinematic measurements in this study were comparable to those reported in the literature, and no significant differences were found in stride parameters (Lemaire et al., 2000). Kobayashi et al. (2024) found no significant differences in spatiotemporal parameters between subjects using a TFsim and prosthesis users, though walking speed was not reported. The only significant gait difference observed was in vertical GRFs, where there was a significant difference in the first and second GRF peaks. No kinetic or kinematic measurements were performed in this study (Kobayashi et al., 2024). These findings suggest that TFsims can mimic the spatiotemporal parameters of prosthetic gait.

One drawback of TFsims is the presence of the healthy lower leg behind the user. With the knee at an angle of 90 degrees in case of an L-shaped interface, the leg is pointing backwards as far as possible. The lower leg therefore causes an inertial artifact (Vanicek et al., 2007; Sup et al., 2008; Waycaster et al., 2010; Thatte and Geyer, 2015; Schlafly and Reed, 2020), decreasing the moment of inertia of the affected leg relative to the transverse axis through the hip joint. A high mass of the simulator in combination with a dorsal placement of the center of mass will increase this effect. To achieve adequate swing of the affected limb, this increases its angular velocity and can encourage a longer step length, thereby producing and possibly exacerbating gait asymmetry (Vanicek et al., 2007). This effect is confirmed by the study of Bonnet-Lebrun et al., 2024, which found a larger asymmetry in step length in able-bodied individuals walking with a TFsim compared to the reported step length asymmetry in transfemoral prosthetic gait. Kobayashi et al., 2024 also found a longer step length in the affected leg, but this effect did not increase step length asymmetry beyond that observed in transfemoral prosthetic gait. However, Kobayashi et al., 2024 used the V-shaped interface, where holding the lower leg closer to the body might decrease the effect of the inertial artifact. Other characteristics such as comfort and usability cannot be compared between L-shaped and V-shaped sockets since no literature is available.

## 4. Discussion

### 4.1. Overview of current simulator configurations

#### 4.1.1. Various prosthesis simulator designs and mounting approaches

This review identified two different mounting approaches (distal mounting approach and lateral mounting approach) and three different amputation levels (simulating a transtibial amputation, a transfemoral amputation, and a hip disarticulation) for able-bodied adapters. Most simulators were designed for simulating a unilateral amputation; for transtibial and transfemoral amputations, simulators for simulating bilateral amputations were also found. In transfemoral amputation simulators, two different designs were identified, a so-called L-shaped simulator in which the knee is flexed 90 degrees and a so-called V-shaped simulator in which the knee is flexed maximally.

#### 4.1.2. Materials and mass characteristics

A few articles described the material used to construct the simulators and their overall mass. Simulators included plastics and metals, sometimes already dictated by the commercial device that had been adapted for use as a simulator. Most articles did not mention material and mass properties, making it hard to compare the properties of simulators.

#### 4.1.3. Impact of configurations on biomechanics and gait simulation

Prosthesis simulators with a lateral configuration can theoretically address some of the issues of distal configuration simulators by minimizing leg length discrepancy and aligning the prosthesis center of rotation with that of the anatomical joints (Flowers, 1973; Hoover and Fite, 2010; Waycaster et al., 2010). Aligned joint centers may be useful for TFsims, since asymmetric knee center heights may result in extended load acceptance time (Schlafly and Reed, 2020), although this behavior would also be influenced by the compliance of the simulator and attached prosthesis. The obvious drawback of a TFsim lateral configuration is the imposition of a frontal plane moment due to limb lateral offset (Sup et al., 2007), which has been reported to cause user discomfort (Sup et al., 2008).

Similarly, a HDsim lateral configuration has been reported to cause a cantilever effect on the prosthesis as its longitudinal axis is not aligned with the gravitational vector acting on the center of mass. This configuration may produce loads within the prosthesis that do not represent those during prosthetic gait (Bader et al., 2023). While the lateral configuration of HDsims might alter the gait of able-bodied individuals, including balance metrics due to the widened base of support (Waycaster et al., 2010), no conclusive evidence has been reported (Bader et al., 2023).

### 4.2. Advantages and potential of prosthesis simulators – why simulators offer a promising path in prosthesis development

#### 4.2.1. Role of simulators in addressing challenges associated with testing

In the development of leg prosthesis, iterative testing is crucial. Leg prosthesis simulators can help advance prosthesis development without putting a high burden or extra risk on leg prosthesis users. Simulators can also advance leg prosthesis development, as the ethical approval processes for testing with able-bodied individuals are more time-efficient than the medical ethical approval process for testing with prosthesis users, resulting in earlier preliminary testing and more design iterations.

#### 4.2.2. Opportunities for research, device testing, and training

The articles in this review show that able-bodied adapters can play a key role in preliminary testing of new prosthesis designs and control strategies. Prosthesis simulators are also used to evaluate the effectiveness of the newly designed prosthesis on biomechanical factors such as gait performance although their effects on the actual user are poorly understood. A last use case can be to use simulators for training therapists in aligning and adjusting prostheses (Kobayashi et al., 2024).

#### 4.2.3. Design criteria for future prosthesis simulators

Unfortunately, there is no golden standard for designing prosthesis simulators. In a wide variety of testing applications, prosthesis designs, and levels of amputation, there is not one simulator design that can be marked as optimal. However, this review identifies a set of four design criteria that can minimize compensation and allow able-bodied users to better simulate prosthetic gait. To improve the gait characteristics of prosthesis simulators, the following design guidelines can be followed:

1. A low leg length discrepancy, preferably below 2 cm.
2. Alignment of non-affected and prosthetic joints where possible.
3. A lightweight socket design with the center of mass of the socket close to the simulated amputated joint.
4. A minimal frontal plane moment, preferably with load lines crossing the non-affected joints on the amputated side.

Although generally formulated, the authors think that the mentioned guidelines can minimize compensation strategies and improve the prosthetic gait of able-bodied users. However, optimization comes with a trade-off. The optimal design is dependent on the objective of the research and the use of the simulator. The four mentioned design criteria always need to be weighed against the objective of the simulator to achieve an optimal design.

### 4.3. Current challenges with simulators

#### 4.3.1. Leg length discrepancy issues and related biomechanical consequences

Prosthesis simulators have the effect of increasing the length of the affected leg, which then requires either bilateral simulator use or adjustments to the unaffected leg to address the leg length discrepancy. Thirty-five articles, with both distal and lateral configurations, have mentioned this adjustment or bilateral use, while the remaining have made no mention of an accommodation and may therefore have imposed a leg length discrepancy. Asymmetry in leg length up to 2 cm is not uncommon in able-bodied persons (Knutson, 2005), while asymmetries larger than 2 cm can lead to altered kinematics (Vanicek et al., 2007). The study by Murabayashi and Inoue, 2020 imposed a leg length discrepancy of 6.6 cm with the use of a TFsim, which the authors believe generated a later onset of hip flexion in stance (60% of the gait cycle compared to the typical 50%).

Apart from leg length discrepancy, increased leg length can also produce increased joint moments. In the study of Grimmer et al., 2016, the affected leg was approximately 3.5 cm longer when wearing a TTsim than the unaffected leg, which exhibited increased vertical GRF peaks that resulted in higher ankle torques in the unaffected limb. Although the increased loads may be attributed to leg length discrepancy and falling onto the unaffected limb, the study by Jin et al., 2016 addressed this discrepancy when using a TFsim with a lift shoe and still observed increased unaffected limb ankle and knee joint moments.

#### 4.3.2. Other limitations: inertial artifacts, comfort, and altered joint mechanics

The added mass of prosthesis simulators, including the attached prosthesis, has been reported to increase metabolic demands (Adamczyk et al., 2006; Caputo and Collins, 2014; Kim and Collins, 2015; Pillet et al., 2022). An added mass of 2% body mass to the affected limb can increase the metabolic rate by up to 20% (Caputo and Collins, 2014). Every kilogram of mass added to the ankle can increase the metabolic rate by 11–24%, partly depending on the location of the (center of) mass (Adamczyk et al., 2006). Collins and Kuo, 2010 reported an unexpected increase in positive work performed by the sound limb and stated the added mass of the prosthesis simulator as a possible cause. Considering the effect of mass on metabolic demands, it seems reasonable to use the lightest possible materials while still being able to withstand the necessary loads during operation. Articles described TTsims being used, by participants with a mass up to 95 kg for walking (Gholizadeh et al., 2024) or 85 kg with a maximal running speed of 10 km/h (Groothuis and Houdijk, 2019). Moreover, HDsims were used, by subjects with a reported mass up to 100 kg (Mroz et al., 2024).

### 4.4. Training and accommodation considerations

#### 4.4.1. Variability of training protocols

In two articles, a gait evaluation with the TFsims was performed to compare kinematic, kinetic, and spatiotemporal parameters of participants walking with a prosthesis simulator with those of prosthesis users (Lemaire et al., 2000; Kobayashi et al., 2024). To enhance the internal validity of such a comparison, simulator users should be experienced and able to walk comfortably. However, there was an inconsistency in the definition of “sufficient” training. While both articles thoroughly trained subjects, Kobayashi et al., 2024 only included participants who had used the TFsim for over 6 months, while Lemaire et al., 2000 trained their participants in two gait training sessions of 45–60 minutes under the guidance of a physiotherapist.

Another example of uncertainty surrounding sufficient training with prosthesis simulators was observed for running with a simulator. Groothuis and Houdijk, 2019 provided their participants with two practice sessions to learn how to run with a TTsim. The authors mentioned that even though participants felt comfortable running, they believed that more training was required to master this skill. A total of three articles included running in their experiments, but only Groothuis and Houdijk, 2019 mentioned participant experience. It is therefore difficult to infer the amount of training time that should be applied to obtain reliable results for running with a prosthesis simulator.

#### 4.4.2. Importance of training in achieving realistic gait patterns

TFsims are used for research on adaptation to novel situations in two articles (Vanicek et al., 2007; Kooiman et al., 2020). Vanicek et al., 2007 reported a linearly increasing walking speed during the first 20 minutes of walking with the prosthesis simulator after fitting, which then plateaued. Participants were immediately able to walk without assistance. This finding is consistent with that of Kooiman et al., 2020, where the gait cycle duration converged toward normal after 12 minutes of walking. Although these findings indicate that subjects may acclimate to the prosthesis simulator to the point of unaided ambulation, it does not necessarily imply that participants will adopt a gait pattern most closely aligned with either their natural gait or those of prosthesis users of which they are simulating, but rather some unique “prosthesis simulator gait.”

Importantly, a lack of training can influence the gait performance of simulator users. Liu et al., 2013 reported that a walking speed of 0.6 m/s was difficult to reach for untrained subjects when using a TFsim, while participants who received training could exceed this speed.

#### 4.4.3. Lack of standardization in accommodation procedures

As mentioned in Section 4.4.2, there is considerable variance in the amount of training and accommodation with prosthesis simulators prior to testing. This lack of consensus induces uncertainty about the generalization and comparison of the results of this review.

### 4.5. Performance activities and functional limitations

#### 4.5.1. Types of movements successfully tested (walking, running, stair ascent, etc.)

One of the primary objectives of this review was to assess the utility of prosthesis simulators, which includes the range of possible performance activities that can be achieved with their use. The literature suggests that activities including walking in steady and non-steady environments across different surface gradients and completing transitions such as sit-to-stand and stair ascent are possible.

#### 4.5.2. Inconsistencies and mixed findings regarding feasibility of certain activities

Across studies with different performance activities, some mixed results were found. Andrysek et al., 2019 evaluated gait for a transfemoral prosthesis user and an able-bodied participant using a TFsim. While the prosthesis user was evaluated walking at various speeds, on slopes, stairs, and navigating obstacles, the able-bodied participant could only complete walking at a self-selected speed due to their limited mobility with the simulator. However, other reviewed TFsim articles reported that those other activities could be completed, suggesting possible inconsistencies in either experimental protocols or participant capabilities. For instance, the study by Murabayashi and Inoue, 2020 designed a locking mechanism for running with a prosthetic knee, but the able-bodied participant was unable to run due to insufficient skill, as suggested by the authors, whereas another study demonstrated the feasibility of running with a TFsim (Zhu et al., 2022).

#### 4.5.3. Need for standardized testing protocols for evaluating performance

Overall, the examples in Section 4.5.2 underscore the uncertainty in the literature regarding the full range of performance activities possible with lower limb prosthesis simulators, highlighting the need for systematic evaluations and standardized testing protocols.

### 4.6. Recommendation for future research and development

More research is needed to better understand the capabilities and effects of lower limb prosthesis simulators on the testing of prosthetic components during ambulation and their ability to replicate prosthetic gait to be useful for prosthetic research.

Although the influence of TFsims on gait has been evaluated (Lemaire et al., 2000; Kobayashi et al., 2024), similar evaluations for TTsims and HDsims are lacking. As a result, it remains unclear which gait parameters are affected by these simulators and to what extent they differ from actual prosthesis use. Additionally, the range of performance activities for each type of simulator and each configuration should be evaluated. Lastly, the amount of training necessary for different movements should be evaluated for all three types of simulators and the configurations of the prosthesis simulators.

A last recommendation is to include the definition of a prosthesis simulator in the ISPO dictionary (Dictionary – ISPO, 2025). Figure 3 shows that different terms are used for the same type of devices, and the authors propose to use the term “prosthesis simulator” and to include this term in the ISPO dictionary.

### 4.7. Study limitations

One limitation of the reviewed literature was the absence of detailed descriptions of prosthesis simulators in several studies. In those cases, relevant information had to be extracted from figures or supplementary videos, when available, rather than from the main text. Despite efforts to gather the most accurate information possible, some details may remain incomplete.

An additional limitation concerns the possibility that not all relevant studies were identified. This is due to the wide variety of terms used to describe prosthesis simulators and to the fact that their use is often not explicitly mentioned in abstracts. As a result, some studies may have been overlooked. However, given the substantial number of studies included, it is unlikely that additional studies would alter the overall observations and conclusions.

Also, the literature was retrieved from only a limited number of databases. And the inclusion and exclusion criteria for each article were only checked by two reviewers.

Finally, the heterogeneity in study designs, subject populations, and methodologies renders direct comparisons challenging. Differences in training protocols, prosthesis simulator configurations, and outcome measures limit the ability to draw generalized conclusions. However, the large number of reviewed studies that spanned decades helps engender confidence in the results.

## 5. Conclusion

Iterative testing is crucial for the development of lower limb prostheses. However, conducting research with individuals with limb loss poses challenges such as medical approval processes and participant recruitment. Prosthesis simulators, which enable able-bodied individuals to use lower limb prostheses, provide a viable alternative for preliminary testing, thereby accelerating the development of new prostheses. This is the first review to provide a structured overview of the different types of lower limb prosthesis simulators, their applications, and the factors influencing their utility. By identifying inconsistencies in training protocols, performance capabilities, and reporting standards, this review highlights areas where future research is needed to improve standardization and comparability between studies. This review included 73 studies using a simulator. Three different types of prosthesis simulators were identified: transfemoral, transtibial, and hip disarticulation simulators. Lower limb prosthesis simulators were mainly utilized for testing new leg prosthesis designs and control mechanisms. While prosthesis simulators appear valuable for these purposes, their effects on user performance remain poorly understood. The literature assessing the effect of simulator use on gait was limited mainly to TFsims, whereas similar evaluations were lacking for TTsims and HDsims. Furthermore, most studies focus on walking, while other activities were assessed far less frequently, making it unclear whether simulators are inherently limited to walking or other activities are simply understudied. Training on prosthesis simulators prior to testing may also have an important influence on performance outcomes, but there was little consensus on the amount of training required for meaningful results. Despite these research gaps, lower limb prosthesis simulators have the potential to accelerate prosthesis research and development. This potential is established by gait evaluations of TFsims, which demonstrated only small differences compared to the gait of transfemoral prosthesis users, as well as the ability of lower limb prosthesis simulators to accommodate a wide range of activities. An improved understanding of the effects of simulator design and training could improve research reliability and enhance prosthesis design and user adaptation.

## Data Availability

This literature review was conducted using publicly available research articles. No new data were generated or analyzed for this study.
